# Supplementary material for: Evolution of Antimicrobial Susceptibility to Penicillin in Invasive Strains of Streptococcus pneumoniae during 2007–2021 in Madrid, Spain
Source: Antibiotics (Basel). 2023 Feb 1;12(2):289. doi: 10.3390/antibiotics12020289 (PMC9952450; doi:10.3390/antibiotics12020289)
Supplement: Supplementary file 1 [file antibiotics-12-00289-s001.zip › antibiotics-2161995-supplementary.pdf]

Supplementary File

Table S1. CLSI and EUCAST MIC breakpoints.

	**Penicillin CMI (µg/ml)**		
	***S**	***I**	**R**
CLSI MIC breakpoints criteria (M100 30th Edition year 2020)			
CLSI MIC breakpoints (parenteral non-meningitis)	≤2	4	≥8
CLSI MIC breakpoints (parenteral meningitis)	≤0.06		>0.12
CLSI MIC breakpoints (oral penicillin V)	≤0.06	0,12- 1	≥2
EUCAST MIC breakpoints criteria (Edition 2021)			
EUCAST MIC breakpoints (indications other than meningitis)	≤0.06		>2
EUCAST MIC breakpoints (for meningitis)	≤0.06		>0.06
